# Statistical Analysis of Graph-Theoretic Indices to Study EEG-TMS Connectivity in Patients With Depression

**DOI:** 10.3389/fninf.2021.651082

**Published:** 2021-04-08

**Authors:** Elzbieta Olejarczyk, Adam Jozwik, Vladas Valiulis, Kastytis Dapsys, Giedrius Gerulskis, Arunas Germanavicius

**Affiliations:** ^1^Nalecz Institute of Biocybernetics and Biomedical Engineering, Polish Academy of Sciences, Warsaw, Poland; ^2^Faculty of Physics and Applied Informatics, University in Łódź, Łódź, Poland; ^3^Life Sciences Center, Institute of Biochemistry, Vilnius University, Vilnius, Lithuania; ^4^Republican Vilnius Psychiatric Hospital, Vilnius, Lithuania

**Keywords:** transcranial magnetic stimulation, graph-based EEG connectivity, depression, directed transfer function, k-NN rule based statistical analysis

## Abstract

**Aim:**

The objective of this work was to demonstrate the usefulness of a novel statistical method to study the impact of transcranial magnetic stimulation (TMS) on brain connectivity in patients with depression using different stimulation protocols, i.e., 1 Hz repetitive TMS over the right dorsolateral prefrontal cortex (DLPFC) (protocol G1), 10 Hz repetitive TMS over the left DLPFC (G2), and intermittent theta burst stimulation (iTBS) consisting of three 50 Hz burst bundle repeated at 5 Hz frequency (G3).

**Methods:**

Electroencephalography (EEG) connectivity analysis was performed using Directed Transfer Function (DTF) and a set of 21 indices based on graph theory. The statistical analysis of graph-theoretic indices consisted of a combination of the *k*-NN rule, the leave-one-out method, and a statistical test using a 2 × 2 contingency table.

**Results:**

Our new statistical approach allowed for selection of the best set of graph-based indices derived from DTF, and for differentiation between conditions (i.e., before and after TMS) and between TMS protocols. The effects of TMS was found to differ based on frequency band.

**Conclusion:**

A set of four brain asymmetry measures were particularly useful to study protocol- and frequency-dependent effects of TMS on brain connectivity.

**Significance:**

The new approach would allow for better evaluation of the therapeutic effects of TMS and choice of the most appropriate stimulation protocol.

## Introduction

Some psychiatric diseases, like depression, may be characterized as dysconnectivity disorders (Friston, 1996; Schmitt et al., 2011). The missing connections can be rebuilt via neuroplasticity (Noda et al., 2018; Wilson et al., 2018; Iglesias, 2020; Zrenner et al., 2020), which refers to the ability of the brain to change over the course of the lifespan (Pascual-Leone et al., 2011; Zappasodi et al., 2015). Neuroplastic changes can be observed under different stimuli or during task performance (Cottone et al., 2017; Olejarczyk et al., 2017), and in various disease states such as Alzheimer’s disease (Ahmadlou et al., 2010; Morabito et al., 2015), depression (Zuchowicz et al., 2019; Olejarczyk et al., 2020), schizophrenia (Olejarczyk et al., 2017; Yuchao et al., 2019), Parkinson’s disease (Galvez et al., 2018), stroke (Zappasodi et al., 2014), and epilepsy (Jiang et al., 2018).

Transcranial magnetic stimulation (TMS) is a non-invasive neuromodulation technique that is applied as an alternative therapy in drug-resistant patients to restore impaired neural connections. The combination of TMS and electroencephalography (EEG) allows for the non-invasive study of changes in brain connectivity (Shafi and Pascual-Leone, 2012).

Recent studies examining connectivity using EEG data recorded in patients with depression have predominantly used non-directional measures (i.e., coherence; Leuchter et al., 2012), various measures of phase synchronization (e.g., Phase-Locking Value; Zuchowicz et al., 2019), Phase Lag Index (Olbrich et al., 2014), Katz’s and Higuchi’s fractal dimensions, and other non-linear methods or their combinations (Acharya et al., 2015). The application of directional measures, such as Directed Transfer Function (DTF) (Kaminski and Blinowska, 1991), allows for better understanding of the mechanisms involved in neural network re-organization associated with changes in brain states in different conditions among healthy persons or patients suffering from psychiatric disorders. To our knowledge, only one study has applied DTF and graph-theoretic indices to study the effects of TMS on functional connectivity in depression using a standard stimulation protocol, i.e., 10 Hz repetitive TMS (rTMS) applied over the left dorsolateral prefrontal cortex (DLPFC) (Olejarczyk et al., 2020). Other stimulation protocols have been used in only a few other studies. In particular, previous research has shown that the application of 1 Hz rTMS over the right DLPFC causes a shift in frontal alpha power asymmetry toward the right hemisphere (Valiulis et al., 2012).

Most prior studies have performed statistical analyses on indices based on graph theory using analysis of variance (ANOVA) or a non-parametric test equivalent. Some authors have applied various classifiers to differentiate between patients with depression and controls by applying a combination of different non-linear connectivity methods to extract features from EEG signals (Acharya et al., 2015). However, to-date, none of the classifiers used in statistical analyses have utilized a wide range of graph-theoretic indices derived from the adjacency matrices of a connectivity measure.

In the present study, we compared the results of the connectivity analysis evaluated by DTF and graph-theoretic indices using three different TMS protocols: (1) 10 Hz rTMS over the left DLPFC, (2) 1 Hz rTMS over the right DLPFC, and (3) intermittent theta burst stimulation (iTBS). Moreover, we applied a novel approach to the statistical analysis of graph-theoretic indices using the *k*-NN rule combined with the leave-one-out method and a statistical test using a 2 × 2 contingency table. This approach is novel because it calculates the significance level for the dependence between the class label and a feature vector to evaluate whether this dependence exists at all. This is particularly important in the case of high misclassification rates, which is relevant for the present study.

The primary objective of this work was to apply a novel method of statistical analysis (Harnisz et al., 2020). The usefulness of this method was demonstrated by analyzing the impact of various TMS protocols on EEG connectivity patterns in patients with depression. The advantage of the proposed method is the ability to identify statistically significant differences between conditions or stimulation protocols using a vector comprised of the optimal combination of features found among all analyzed features. This approach is in contrast to non-parametric tests, which rely on a single feature.

## Materials and Methods

### Patient Recruitment and Diagnosis Based on Psychiatric Tests

EEG data were collected in the Republican Vilnius Psychiatric Hospital (RVPH) following approval by the local ethics committee. TMS was applied to treat patients who were diagnosed with drug-resistant depression. One hundred twenty-six patients with a drug-resistant depressive disorder were recruited from the RVPH. Eligible participants ranged in age from 18 to 75 years. Participants were split into three treatment groups, and demographic data for the three treatment groups are reported in Table 1. The three groups consisted of: (1) 35 patients (30 females, 5 males) who underwent 1 Hz rTMS over the right DLPFC, (2) 77 patients (55 females, 22 males) who underwent 10 Hz rTMS over the left DLPFC, and (3) 14 patients (11 females, 3 males) who underwent iTBS. All patients were screened according to the Diagnostic Criteria for Research (DCR) of International Classification of Diseases—tenth edition (ICD-10). All participants provided written informed consent prior to taking part in this study.

**TABLE 1:** Characteristics of the patient groups.

Group	TMS protocol	N (F/M)	Mean ± SD age (years)
G1	1 Hz rTMS over the right DLPFC	35 (30/5)	50.2 ± 10.27
G2	10 Hz rTMS over the left DLPFC	77 (55/22)	50.05 ± 14.18
G3	intermittent theta burst stimulation (iTBS) over the left DLPFC	14 (11/3)	55 ± 14.7

**N, number of patients; F/M, number of females/males; G1, G2, G3, indicate the TMS stimulation protocols.**

Inclusion criteria for patients with depression were as follows: (a) history of at least one previous affective episode, according to the Diagnostic Criteria for Research of ICD-10, and (b) a total score greater than 18 on the 17-item Hamilton Depression Rating Scale (HAM-D; Hamilton, 1960), and (c) resistance to at least two different antidepressant medications or their combinations. There were no gender restrictions; however, we did aim to achieve a balance of genders during recruitment. Exclusion criteria were as follows: (a) history of medical condition(s) that could entail cognitive deterioration, (b) history of epilepsy, (c) recent drug or alcohol abuse, (d) pregnancy, (e) diagnosis of a learning disability, (f) inability to understand or complete the cognitive assessment, or (g) any other contraindication(s) for TMS. After participants were enrolled into the study, previously administered antidepressant treatments were maintained. However, psychotropic medications that can affect the stimulation threshold—particularly benzodiazepines—were tapered. Psychotropic drug doses were required to be stable for at least 2 weeks prior to initiating the first TMS session and for the entire duration of the treatment.

### TMS

TMS procedures were applied using the MagVenture Magpro X100 TMS stimulator with the MagVenture Cool Coil B65 liquid cooled figure-eight coil. 280 μs biphasic impulses were used during the stimulation.

TMS is a broad term that includes different types of stimulations, such as single pulse stimulation for motor threshold evaluation or therapeutic rTMS. iTBS is one type of rTMS protocol (Schwippel et al., 2019). As a comparison to a commonly used 10 Hz rTMS (G2), we applied two other protocols: iTBS over the left DLPFC (G3), and 1 Hz rTMS over the right DLPFC (G1). Physiologically, iTBS is expected to facilitate the activity of the stimulation site by delivering 20 excitatory stimulation trains, which is similar to the 10 Hz condition. However, iTBS is expected to facilitate this activity in a shorter time period as compared to the 10 Hz condition (i.e., 3 min vs. > 15 min). A shorter duration of stimulation, and thus more comfort to the patient, is the main advantage of this protocol.

The intensity of rTMS was set according to the resting motor threshold. The resting motor threshold was defined as the lowest intensity needed to generate a visible twitch of the thumb of the participants’ relaxed hand. Stimulation intensities were set at 100% for high frequency rTMS over the left DLPFC, 120% for low frequency rTMS over the right DLPFC, and 80% for iTBS rTMS over the left DLPFC. All rTMS frequencies greater than or equal to 5 Hz are considered high frequency (HF), whereas all rTMS frequencies less than or equal to 1 Hz are considered low frequency (LF).

HF rTMS consisted of twenty 10 Hz stimulation trains that each lasted 8 s and were spaced at 40-s intervals (1,600 impulses in total) (Rossi et al., 2009). LF rTMS consisted of 1 Hz stimulation in 2 trains, each lasting for 60 s, and spaced at 3-min intervals (120 impulses in total) (Klein et al., 1999). iTBS consisted of 2 s 50 Hz three-pulse bursts that were presented at 5 Hz frequency, applied in 20 trains and spaced at 8-s intervals (600 impulses in total) (Huang et al., 2011; Schwippel et al., 2019). The stimulation site was chosen by the psychiatrist according to the prevailing symptoms of depression. In particular, the left DLPFC was chosen in the case of adynamic depression, i.e., depression with dominating apathy symptoms, whereas the right DLPFC was chosen in case of anxious depression (Neznamov et al., 2001; Hunter et al., 2019; Minzenberg and Leuchter, 2019; Zorzo et al., 2019; Balderston et al., 2020). Right DLPFC activity seems to be directly linked to the manifestation of anxiety (Balderston et al., 2020). Indeed, 1 Hz rTMS over the right DLPFC is often used to treat anxious depression, as well as anxiety disorders and posttraumatic stress disorder (PTSD) (Zorzo et al., 2019). The HF rTMS and iTBS stimulation protocols were selected randomly. The imbalance in number of participants across the two stimulation sites (see Table 1) is due to practical limitations. In particular, the duration of classical 1 Hz protocol is shorter than the 10 Hz protocol, and thus does not require a shortened TBS alternative. All patients underwent 10–30 procedures.

The Localite TMS Navigator MR-less system was used for coil placement. This neuronavigation system utilized a standard Montreal Neurological Institute (MNI) map with targets placed at the following coordinates: (1) x = −41, y = 16, z = 54 for left DLPFC; (2) x = 40, y = 48, z = 35 for right DLPFC (Teneback et al., 1999; Valiulis et al., 2012; Fox et al., 2013).

### EEG Registration and Preprocessing

EEG signals were recorded for 10 min during an eye-closed resting-state condition prior to rTMS and again 20–30 min after the last rTMS session. EEG data were collected in an electrically shielded booth using EBNeuro Galileo Mizar apparatus. A cap with twenty round bridge type Ag/AgCl electrodes was placed according to the international 10–20 system. The Fpz electrode was used as the ground electrode and ear electrodes were used as the reference. EEG recordings were filtered with low frequency (0.53 Hz), high frequency (70 Hz), and notch (50 Hz) filters. Data were digitized at 256 Hz at 12 bits. For further analysis, 30 s EEG intervals without artifacts before and after stimulation in all 126 patients with depression were chosen by an expert. In this study we decided to perform rather the analysis of between-subject than within-subject effects.

### Connectivity Analysis

The DTF is a directed linear connectivity method that is based on Granger Causality but defined in the frequency domain. DTF allows one to determine the localization of sources and the direction of EEG activity propagation (Kaminski and Blinowska, 1991). The model order was estimated using the Akaike information criterion (AIC) defined as follows:

(1) $$A\,I\,C\,\left(d\right)=2*\log\left(\det\left(V\right)\right)+\frac{2\,k\,d}{N}$$

where V is the noise variance matrix, N is the window size, d is the model order, and k is the number of EEG channels.

In the calculation of DTF, the following relation should be satisfied to guarantee the quality of fitting of the model (Blinowska and Kaminski, 2006): k ^∗^ d < 0.1 ^∗^ N. In the present study, the model order d was set to 10, the number of EEG channels k was 20, and the window size was 256 Hz ^∗^ 30 s = 7,680 samples. Thus, the above relation was satisfied. The DTF was calculated using HERMES software (Niso et al., 2013)^1^. The significance of DTF values was determined using surrogate data analysis. The amplitudes of signal were maintained, while the frequency relationships were modified by shuffling of the data in frequency domain. The signal values were then obtained again from surrogate data that was transformed to the time domain. Significance of the values was evaluated by comparing the results from the original signal with results obtained from surrogate data. The number of surrogates was set to 100. Adjacency matrices were obtained from data with a *p*-value below the 0.05 threshold.

The matrices were analyzed using graph-theoretic indices. All indices derived from matrices of adjacency for DTF were calculated independently for each of the 126 patients separately for five frequency bands and for 10 thresholds, i.e., 10 values obtained from the matrices that contained 0–90% of the strongest connections. The indices dependent on the EEG channel were averaged across all twenty channels.

A disadvantage of the analysis of graph-theoretic indices is their dependence on *degree* index. Results are typically reported as graphs of index values as a function of the threshold (Olejarczyk et al., 2017; Olejarczyk and Jernajczyk, 2017), or as index values averaged across all thresholds (Olejarczyk et al., 2020). In this paper, we propose to include the values of indices calculated for each of 10 thresholds in the classification step instead to take an average value. The *k*-NN classifier can automatically select the thresholds for which the indices yield the best classification results. However, frequently, an average threshold does not allow for the separation of two populations despite the presence of significant differences between indices for some of thresholds. This may also occur if only an increasing or decreasing trend exists between indices across a larger range of thresholds.

The DTF was calculated in each patient from three 30-s EEG segments within the entire frequency band. Then, adjacency matrices were identified for each frequency band (i.e., delta: 1–3 Hz; theta: 4–8 Hz; alpha: 9–12 Hz; beta: 13–30 Hz; gamma: 31–70 Hz), separately. The weighted matrices of significant connections were analyzed using graph-theoretic indices (Rubinov and Sporns, 2010).

The following graph-based indices were calculated using MATLAB functions provided in the Brain Connectivity Toolbox^2^ : (1) basic measures (i.e., *density*, *degree* and *strength*); (2) measures of integration (i.e., *characteristic path length* and *global efficiency*); (3) measures of segregation (i.e., *clustering coefficient*, *local efficiency* and *modularity*); (4) measures of centrality (i.e., *betweenness centrality*); (5) measures of resilience (i.e., five assortativity coefficients: *add, aoi, aio, aoo, aii*) (Newman, 2003; Leung and Chau, 2007; Rubinov and Sporns, 2010; Olejarczyk et al., 2020); and (6) joint degree distribution (i.e., *jod, jid, jbl*) (Olejarczyk et al., 2020).

We also calculated four measures of frontal-posterior and hemispheric asymmetry: (1) frontal-posterior displacement (*DFP*), (2) displacement between the left and right hemispheres (*DLR*), (3) frontal-posterior influence (*IFP*), and (4) influence between left and right hemisphere (*ILR*). These measures were calculated using MATLAB functions developed in-house (Olejarczyk and Jernajczyk, 2017; Olejarczyk et al., 2020). A detailed description of the DTF method and definitions of the graph-theoretic indices is provided elsewhere (Olejarczyk et al., 2020).

### Novel Approach in Statistical Analysis of Graph-Theoretical Indices

We examined differences in the TMS effect between two conditions (i.e., before and after TMS), as well as, differences between the three stimulation protocols (i.e., G1, G2, G3) separately, across five frequency bands.

The error rate estimated by the leave-one-out method was used as a feature selection criterion. The data matrices for the feature selection contained 700, 1,540, and 280 rows for G1, G2, and G3, respectively. These sizes were obtained according to the following formula: number of patients × 10 thresholds × 2 conditions. The first column of this matrix contained the class label, which was either the TMS condition (pre, post) or the protocol number (G1, G2, G3). Next, there were 21 columns that corresponded to each of the 21 features.

We proposed a novel approach in the statistical analysis of graph-theoretic indices. Our approach combines two methods. One method was adapted from an area known as Pattern Recognition, and the other was a non-parametric test based on a 2 × 2 contingency table: chi-square, V-square, chi-square with Yates correction, or Fisher’s exact test (Stanisz, 2006). To form the 2 × 2 contingency table, we used the *k* nearest neighbor (*k*-NN) rule, which is the most popular method in Pattern Recognition (Cover and Hart, 1967). The *k*-NN rule is also considered to be one of the most powerful rules and offers very good performance (Carpenter and Grossberg, 1996).

The *k*-NN rule is simple to implement and does not require a training session if the feature set is established. The main disadvantage of the *k*-NN rule is a slow classification phase. The *k*-NN rule is used in the classification of objects, described by some features that assume numerical values. A *k*-NN classification rule (or “classifier”) is based on a reference set that contains objects with known class membership. The object to be classified can be any object that is outside of the reference set. This object is assigned to the class that is most heavily represented from among its *k* nearest neighbors in the reference set, for example, using a measure of Euclidean distance. The value of *k* can be established experimentally or taken as a small natural number. In the present study, we assumed *k* = 3, as suggested by Wilson (1972). An ideal classifier is a very rare case, and we need to account for potential misclassifications. In 1965, Lachenbruch (1965) first described the leave-one-out method—a method for estimating the error rate in small data sets. This technique was also described in 1982 by Devijver and Kittler (1982). The leave-one-out method allows for the classification of each object, *x*, in the reference set, R, using the *k*-NN rule, which is based on a reference set R-{*x*}, i.e., the reference set minus the object being currently classified. If for *r* objects the assigned class differs from the true one, then an error rate will be *err* = *r*/*N*, where *N* denotes a number of objects in the reference set. In addition to calculating the error rate, a confusion matrix (defined in Table 2) can be computed.

**TABLE 2:** The confusion matrix.

Contingency table	Assigned to class 1	Assigned to class 2
True class 1	r11	r12
True class 2	r21	r22

The confusion matrix is typically used to calculate sensitivity and specificity of the classification. In our case, the confusion matrix is used to calculate the significance level using the chi-square test or one of its modifications. Recommendations for choosing the correct modification can be found in the Statistica software manual (Stanisz, 2006). The significance level calculated on the basis of the aforementioned contingency table concerns the dependence between two qualitative variables: the true class and the assigned class. Both variables (i.e., the true class and the assigned class) assume values of either 1 or 2. Generally, the assigned class represents the “voice” of features. Thus, the obtained significance level can be treated as concerned to the dependence of the true class on the analyzed set of features.

Not all features can be easily matched to available classes. Some features can be redundant or correlated, which can lead to an increased error rate. Therefore, it is reasonable to perform feature selection, which consists of reviewing various feature combinations. There are two main feature selection procedures: (1) forward feature selection and (2) backward feature selection. The former consists of choosing a single feature that offers the lowest error rate and then sequentially adding additional features if the error rate subsequently decreases. The latter starts with a complete set of features and sequentially rejects the single feature if this action results in a decrease in the misclassification rate. In the present study, we applied the forward feature selection procedure.

**FIGURE 1:**
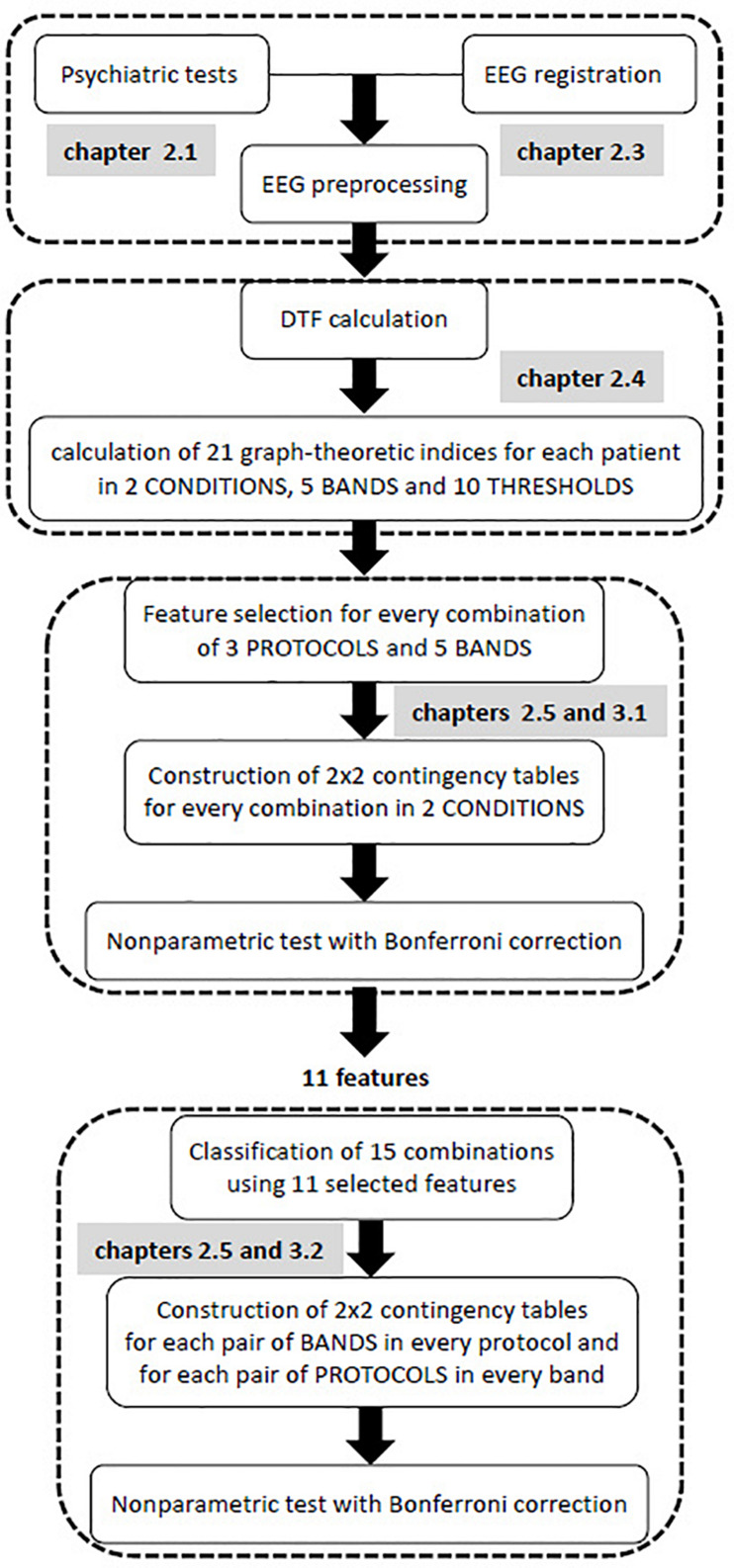
Flow chart of all stages of EEG analysis.

All steps of the data analysis are shown in the flow chart provided in Figure 1. The first step of the statistical analysis was to select the best set of features for each of the 15 classes that were formed from all possible combinations of the 3 protocols and 5 frequency bands. Then, an appropriate non-parametric test with Bonferroni correction based on 2 × 2 contingency table was performed to test for statistically significant differences between two conditions (i.e., pre- and post-TMS) for vectors of selected features in each of these combinations.

The purpose of the second step was to find possible interactions between the 15 classes that were previously considered. Non-parametric tests, such as Kruskal-Wallis or Mann-Whitney U tests, are equivalent to a one-way analysis of variance for independent variables. Therefore, these non-parametric tests allow for the comparison between different pairs of protocols for a given band, or different pairs of bands for a given protocol. In contrast to non-parametric tests which analyzes individual features, the *k*-NN classification analyzes a vector of features. As a result of the *k*-NN classification, the confusion matrix for 15 classes was obtained using all 11 features that were selected in the first step of analysis, i.e., all features that appeared in Table 3. Both conditions (i.e., pre- and post-TMS) were considered in this classification due to the relatively limited dataset. Then, an appropriate non-parametric test with Bonferroni correction was applied based on 2 × 2 contingency table. The non-parametric test aimed to identify statistically significant differences between each pair of bands in each stimulation protocol, and each pair of protocols in every frequency band relative to the set of 11 features.

**TABLE 3:** The results of feature selection and classification by condition (i.e., before and after TMS) performed separately for each of the three stimulation protocols and five frequency bands.

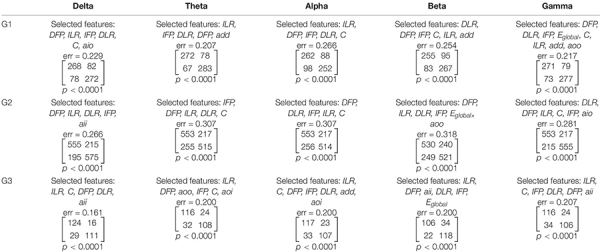

## Results

### Feature Selection Results

The feature selection and condition (i.e., before and after TMS) classification was performed for every pair of stimulation protocols (G1, G2, G3) and frequency bands (δ, θ, α, β, γ). Altogether, 11 of the 21 features were selected, including the *asymmetry measures* (*DFP, IFP, DLR, ILR*), *clustering coefficient* (C), *global efficiency* (*E*_*global*_), and the *assortativity coefficients* (*add, aio, aoi, aoo, aii*). The sets of selected features were found independently for every pair of stimulation protocol and frequency band. Each set starts from a feature that offers the lowest error rate. Further features were included in such a way that the new set would allow to reduce maximally the error rate. The selected features, the error rate, the confusion matrix for the factor CONDITION and the corresponding *p*-values are reported in Table 3.

Using the non-parametric tests based on 2 × 2 contingency table, we found *p*-values that were less than 0.0001 for each contingency table presented in Table 3. Due to the observed high error rates (i.e., ranging from 0.161 to 0.318), we decided to investigate whether there was, in fact, a dependence between the class labels and sets of features. Out of 21 features, the forward feature selection required a review of 231 feature combinations. Accounting for Bonferroni correction, the results can be considered statistically significant if *p* < 0.0002 (i.e., 0.05/231). Thus, the sets of selected features allowed for the identification of statistically significant differences between conditions for each stimulation protocol, and in each frequency band. It is worth noting that each of the *asymmetry measures* appeared in sets of selected features for each stimulation protocol in higher frequency bands (i.e., alpha, beta, and gamma). *Clustering coefficient*, in contrast, was only observed in alpha and gamma bands. The sensitivity and specificity of our method ranged from 0.68 to 0.87, and from 0.68 to 0.83, respectively. Sensitivity and specificity for detecting stimulation impact was calculated from the confusion matrices reported in Table 3.

### Classification of Groups

The set of 11 features previously selected were used for the classification of 15 classes that were formed from all possible combinations of the three stimulation protocols and five frequency bands. The results of this classification are reported in Table 4.

**TABLE 4:** The results of classification based on the *k*-NN rule and the leave-one-out method by 15 classes formed from all possible combinations of the three stimulation protocols and five frequency bands.

class		G1					G2					G3				
				
		δ	θ	α	β	γ	δ	θ	α	β	γ	δ	θ	α	β	γ
		1	2	3	4	5	6	7	8	9	10	11	12	13	14	15
	**δ**	**247**	13	7	8	2	259	81	29	27	10	10	2	3	2	0
	**θ**	7	**136**	25	35	9	44	178	104	109	34	2	7	2	7	1
G1	**α**	3	13	**183**	11	27	20	80	213	50	86	0	0	9	0	5
	**β**	6	42	29	**143**	16	43	171	87	112	41	0	2	0	6	2
	**γ**	1	5	10	5	**231**	10	51	121	41	205	0	0	1	1	18
	**δ**	108	20	2	16	5	**1043**	143	55	74	22	34	8	6	4	0
	**θ**	11	73	25	25	10	120	**776**	207	209	49	2	12	3	17	1
G2	**α**	3	23	80	7	33	65	225	**774**	122	181	1	0	9	5	12
	**β**	10	66	27	47	18	110	394	235	**516**	87	0	10	3	10	7
	**γ**	2	12	19	4	113	28	92	252	102	**864**	0	0	7	2	43
	**δ**	21	4	0	2	1	128	24	11	13	0	**69**	6	0	1	0
	**θ**	8	18	3	4	4	22	84	27	49	10	3	**39**	3	5	1
G3	**α**	3	5	20	3	6	17	34	85	18	41	0	1	**38**	6	3
	**β**	5	15	4	11	3	13	76	32	56	16	1	6	9	**32**	1
	**γ**	0	1	5	1	15	2	20	43	26	116	0	1	0	0	**50**

*The values on the diagonal are marked in bold.*

The values reported in Table 4 were used to construct 2 × 2 contingency tables for every possible pair of frequency bands in each stimulation protocol, and for every possible pair of stimulation protocols in each frequency band. The 2 × 2 contingency tables tested for statistically significant differences between bands for individual stimulation protocols, and for differences between protocols in each frequency band. The observed *p*-values were less than 0.0001 for each contingency table. Thus, there were statistically significant differences between each pair of bands in each stimulation protocol, as well as, between each pair of protocols in each frequency band.

## Discussion

The published literature predominantly applies ANOVA or non-parametric equivalents to assess indices based on graph theory. However, ANOVA assumes that the variables are normally distributed and if not, non-parametric tests are applied. Non-parametric tests are equivalent to a one-way ANOVA in that a single index can be considered. Here, we observed that a specific set of features (i.e., indices) may allow for better differentiation between conditions as compared to relying on a single feature (Olejarczyk et al., 2012). Two populations may have identical ranges of features. Nevertheless, it is possible to separate these conditions by using a distance between the neighbors as a selection criterion. Moreover, the application of the *k*-NN rule allows for the identification of the best set of features without assuming that the features are normally distributed. We chose the leave-one-out method because it is recommended for limited data sets, such as in the present study. Finally, the confusion matrix and chi-square test (or its modification) were used to calculate a significance level for the dependence between two qualitative variables: the true class and the assigned class. The assigned class represents the “voice” of the feature set. The last step is a completely new and universal approach that can be applied in other situations, such as the normal/Gaussian distribution of features or larger data sets. This approach can also be applied in conjunction with other modern classifiers (Ahmadlou and Adeli, 2010; Rafiei and Adeli, 2017).

There are a lot of connectivity measures and indices based on graph theory (Rubinov and Sporns, 2010; Niso et al., 2013). Their usefulness depends on a particular application. In this study we were interested in finding a set of features (indices) allowing for characterizing the changes in brain connectivity evoked by TMS in patients with depression. We have found that 11 from 21 indices were useful for this purpose. They allowed for differentiation between 15 classes being a combination of 3 protocols and 5 frequency bands. Then, we found a set of the best indices for every class separately. These sets can be used in clinical practice for evaluation of the TMS effect for a given protocol or to find differences between protocols. Interestingly, four asymmetry indices were common for three protocols in higher frequency bands (alpha, beta and gamma). This finding is in line with a known characteristic of depression, i.e., alpha hypoactivity of the left and hyperactivity of the right frontal areas during the resting state (Henriques and Davidson, 1991; Davidson, 1998; American Psychiatric Association, 2013; Allen and Reznik, 2015; Mumtaz et al., 2015; Van der Vinne et al., 2017; Kustubayeva et al., 2020). The results of our study showed that also the fronto-posterior asymmetry is important in depression. Moreover, the therapy effect depends on the protocol choice. Thus, the evaluation of initial state of patient with depression by these indices could be helpful for decision support. However, future studies should be performed for more epochs, in a larger sample size with a more balanced groups of patients to test the usefulness of this new methodological approach in clinical practice.

## Conclusion

Here, we applied a novel approach to perform statistical analysis of graph-theoretic indices using the *k*-NN rule combined with the leave-one-out method and a statistical test for the 2 × 2 contingency table. This method allowed for the selection of the best feature set to study protocol- and frequency-dependent effects of TMS on brain connectivity.

The main purpose of this study was to develop a new method of statistical analysis of graph-based indices derived from adjacency matrices of DTF using EEG data recorded in patients with depression before and after TMS. However, this is a general method that could be applied with other data as well.

## Data Availability Statement

The datasets of this manuscript are not publicly available owing to the policy of Republican Vilnius Psychiatric Hospital. Requests to access the datasets should be directed to VV, vladas.valiulis@gmail.com.

## Ethics Statement

The approval of the protocol was obtained from the Ethics Committee of Republican Vilnius Psychiatric Hospital and a written informed consent was provided by all patients.

## Author Contributions

EO: concept of the study, EEG connectivity analysis, statistical data analysis, and wrote the manuscript. AJ: combination of the *k*-NN classification and the leave-one-out method and a statistical test for 2 × 2 contingency table. VV: TMS application and EEG data acquisition and preprocessing. KD: design of the trial and data interpretation. GG: patients recruitment. AG: critical revision of the manuscript. All authors contributed to the article and approved the submitted version.

## Conflict of Interest

The authors declare that the research was conducted in the absence of any commercial or financial relationships that could be construed as a potential conflict of interest.

## Footnotes

1 http://hermes.ctb.upm.es/

2 http://www.brain-connectivity-toolbox.net

## Abbreviations

TMS: Transcranial Magnetic Stimulation
rTMS: repetitive transcranial magnetic stimulation
HF: high frequency
LF: low frequency
iTBS: intermittent theta burst stimulation
DLPFC: dorsolateral prefrontal cortex
EEG: electroencephalography
DTF: Directed Transfer Function
HAM-D: Hamilton Depression Rating Scale
DCR: Diagnostic Criteria for Research
ICD-10: International Classification of Diseases tenth edition.

## References

[B1] Acharya U. R. Vidya S. Adeli H. Jayashree S. Koh J. E. W. Adeli A. (2015). Computer aided diagnosis of depression using EEG signals. *Eur. Neurol.* 73 329–336. 10.1159/000381950 25997732

[B2] Ahmadlou M. Adeli H. (2010). Enhanced probabilistic neural network with local decision circles: a robust classifier. *Integr. Comput. Aid E.* 17 197–210. 10.3233/ica-2010-0345

[B3] Ahmadlou M. Adeli H. Adeli A. (2010). New diagnostic EEG markers of the Alzheimer’s disease using visibility graph. *J. Neural Transm.* 117 1099–1109. 10.1007/s00702-010-0450-3 20714909

[B4] Allen J. J. Reznik S. J. (2015). Frontal EEG asymmetry as a promising marker of depression vulnerability: summary and methodological considerations. *Curr. Opin. Psychol.* 1 93–97. 10.1016/j.copsyc.2014.12.017 26462291 PMC4599354

[B5] American Psychiatric Association (2013). *Diagnostic and Statistical Manual of Mental Disorders (DSM-5)*, 5th Edn. Arlington, VA: American Psychiatric Publishing, 992.

[B6] Balderston N. L. Beydler E. M. Roberts C. Deng Z. D. Radman T. Lago T. (2020). Mechanistic link between right prefrontal cortical activity and anxious arousal revealed using transcranial magnetic stimulation in healthy subjects. *Neuropsychopharmacology* 45 694–702. 10.1038/s41386-019-0583-5 31791039 PMC7021903

[B7] Blinowska K. J. Kaminski M. J. (2006). “Multivariate signal analysis by parametric models,” in *Handbook of Time Series Analysis: Recent Theoretical Developments and Applications*, eds Schelter B. Winterhalder M. Timmer J. (Wernheim: Wiley – VCH), 373–411. 10.1002/9783527609970.ch15

[B8] Carpenter G. A. Grossberg S. (1996). “Learning, categorization, rule formation, and prediction by fuzzy neural network,” in *Fuzzy Logic and Neural Network Handbook*, ed. Chen C. H. (New York, NY: McGRaw-Hill Series on Computer Engineering), 700.

[B9] Cottone C. Porcaro C. Cancelli A. Olejarczyk E. Salustri S. Tecchio F. (2017). Neuronal electrical ongoing activity as a signature of cortical areas. *Brain Struct. Funct.* 222 2115–2126. 10.1007/s00429-016-1328-4 27803994

[B10] Cover T. M. Hart P. E. (1967). Nearest neighbor pattern classification. *IEEE Trans. Inf. Theory* 13 21–27.

[B11] Davidson R. J. (1998). Anterior electrophysiological asymmetries, emotion, and depression: conceptual and methodological conundrums. *Psychophysiology* 35 607–614. 10.1017/s0048577298000134 9715104

[B12] Devijver P. A. Kittler J. (1982). *Pattern Recognition: A Statistical Approach.* London: Prentice Hall.

[B13] Fox M. D. Liu H. Pasqual-Leone A. (2013). Identification of reproducible individualized targets for treatment of depression with TMS based on intrinsic connectivity. *Neuroimage* 66 151–160. 10.1016/j.neuroimage.2012.10.082 23142067 PMC3594474

[B14] Friston K. J. (1996). Theoretical neurobiology and schizophrenia. *Br. Med. Bull.* 52 644–655. 10.1093/oxfordjournals.bmb.a011573 8949263

[B15] Galvez G. Recuero M. Canuet L. del Pozo F. (2018). Short-term effects of Binaural Beats on EEG power, functional connectivity, cognition, gait and anxiety in Parkinson’s Disease. *Int. J. Neural Syst.* 28:1750055. 10.1142/s0129065717500551 29297265

[B16] Hamilton M. (1960). A rating scale for depression. *J. Neurol. Neurosurg. Psychiatry.* 23, 56–62. 10.1136/jnnp.23.1.56 14399272 PMC495331

[B17] Harnisz M. Kiedrzynska E. Kiedrzynski M. Korzeniewska E. Czatzkowska M. Koniuszewska I. (2020). The impact of WWTP size and sampling season on the prevalence of antibiotic resistance genes in wastewater and the river system. *Sci. Total Environ.* 741:140466. 10.1016/j.scitotenv.2020.140466 32886993

[B18] Henriques J. B. Davidson R. J. (1991). Left frontal hypoactivity in depression. *J. Abnorm. Psychol.* 100 535–545. 10.1037/0021-843x.100.4.535 1757667

[B19] Huang Y. Z. Rothwell J. C. Chen R. S. Lu C. S. Chuang W. L. (2011). The theoretical model of theta burst form of repetitive transcranial magnetic stimulation. *Clin. Neurophysiol.* 122 1011–1018. 10.1016/j.clinph.2010.08.016 20869307 PMC3046904

[B20] Hunter A. M. Minzenberg M. J. Cook I. A. Krantz D. E. Levitt J. G. Rotstein N. M. (2019). Concomitant medication use and clinical outcome of repetitive transcranial magnetic stimulation (rTMS) treatment of Major depressive disorder. *Brain Behav.* 9:e01275. 10.1002/brb3.1275 30941915 PMC6520297

[B21] Iglesias A. H. (2020). Transcranial magnetic stimulation as treatment in multiple neurologic conditions. *Curr. Neurol. Neurosci. Rep*. 20:1. 10.1007/s11910-020-1021-0 32020300

[B22] Jiang S. Luo C. Gong J. Peng R. Ma S. Tan S. (2018). Aberrant thalamocortical connectivity in juvenile myoclonic epilepsy. *Int. J. Neural Syst.* 28 1750034. 10.1142/s0129065717500344 28830309

[B23] Kaminski M. J. Blinowska K. J. (1991). A new method of the description of the information flow in the brain structures. *Biol. Cybern.* 65 203–210. 10.1007/bf00198091 1912013

[B24] Klein E. Kreinin I. Chistyakov A. (1999). Therapeutic efficacy of right prefrontal slow repetitive transcranial magnetic stimulation in major depression: a double-blind controlled study. *Arch. Gen. Psych.* 56 315–320. 10.1001/archpsyc.56.4.315 10197825

[B25] Kustubayeva A. Kamzanova A. Kudaibergenova S. Pivkina V. Matthews G. (2020). Major depression and brain asymmetry in a decision-making task with negative and positive feedback. *Symmetry* 12:2118. 10.3390/sym12122118

[B26] Lachenbruch P. A. (1965). *Estimation of Error Rates in Discriminant Analysis.* Ph.D. dissertation, University of California, Los Angeles, CA.

[B27] Leuchter A. F. Cook I. A. Hunter A. M. Cai C. Horvath S. (2012). Resting-state quantitative electroencephalography reveals increased neurophysiologic connectivity in depression. *PLoS One* 7:e32508. 10.1371/journal.pone.0032508 22384265 PMC3286480

[B28] Leung C. C. Chau H. F. (2007). Weighted assortative and disassortative networks model. *Phys. A* 378 591–602. 10.1016/j.physa.2006.12.022

[B29] Minzenberg M. J. Leuchter A. F. (2019). The effect of psychotropic drugs on cortical excitability and plasticity measured with transcranial magnetic stimulation: implications for psychiatric treatment. *J. Affect. Disord*. 253 126–140. 10.1016/j.jad.2019.04.067 31035213

[B30] Morabito F. C. Campolo M. Labate D. Morabito G. Bonanno L. Bramanti A. (2015). A longitudinal EEG study of Alzheimer’s disease progression based on a complex network approach. *Int. J. Neural Syst.* 25:1550005. 10.1142/s0129065715500057 25655033

[B31] Mumtaz W. Malik A. S. Yasin M. A. M. Xia L. (2015). Review on EEG and ERP predictive biomarkers for major depressive disorder. *Biomed. Signal Proces.* 22 85–98. 10.1016/j.bspc.2015.07.003

[B32] Newman M. E. J. (2003). Mixing patterns in networks. *Phys. Rev. E Stat Nonlin. Soft. Matter. Phys.* 67(2 Pt 2):026126. 10.1103/PhysRevE.67.026126 12636767

[B33] Neznamov G. G. Siuniakov S. A. Teleshova E. S. Dorofeeva O. A. Chumakov D. B. Davydova I. A. (2001). Therapeutic action and efficiency of fevarin (fluvoxamine) in patients with non-psychotic anxious and apathic-adynamic depressions. *Zh. Nevrol. Psikhiatr. Im. S S Korsakova* 101 19–24. 11552628

[B34] Niso G. Bruna R. Pereda E. Gutierrez R. Bajo R. Maestu F. (2013). HERMES: towards an integrated toolbox to characterize functional and effective brain connectivity. *Neuroinformatics* 11 405–434. 10.1007/s12021-013-9186-1 23812847

[B35] Noda Y. Zomorrodi R. Daskalakis Z. J. Blumberger D. M. Nakamura M. (2018). Enhanced theta-gamma coupling associated with hippocampal volume increase following high-frequency left prefrontal repetitive transcranial magnetic stimulation in patients with major depression. *Int. J. Psychophysiol*. 133 169–174. 10.1016/j.ijpsycho.2018.07.004 30318052

[B36] Olbrich S. Trankner A. Chittka T. Hegerl U. Schonknecht P. (2014). Functional connectivity in major depression: increased phase synchronization between frontal cortical EEG-source estimates. *Psychiatry Res.* 222 91–99. 10.1016/j.pscychresns.2014.02.010 24674895

[B37] Olejarczyk E. Jernajczyk W. (2017). Graph-based analysis of brain connectivity in schizophrenia. *PLoS One* 12:e0188629. 10.1371/journal.pone.0188629 29190759 PMC5708839

[B38] Olejarczyk E. Jozwik A. Zmyslowski W. Sobieszek A. Marciniak R. Byrczek T. (2012). Automatic detection and analysis of the EEG sharp wave-slow wave patterns evoked by fluorinated inhalation anesthetics. *Clin. Neurophysiol.* 123 1512–1522. 10.1016/j.clinph.2011.12.017 22300687

[B39] Olejarczyk E. Marzetti L. Zappasodi F. Pizzella V. (2017). Comparison of connectivity analyses methods in EEG during resting state. *J. Neural Eng.* 14:036017. 10.1088/1741-2552/aa6401 28378705

[B40] Olejarczyk E. Zuchowicz U. Wozniak-Kwasniewska A. Kaminski M. Szekely D. David O. (2020). The impact of repetitive transcranial magnetic stimulation on functional connectivity in major depressive disorder and bipolar disorder evaluated by directed transfer function and indices based on graph theory. *Int. J. Neural Syst.* 30:2050015. 10.1142/s012906572050015x 32143550

[B41] Pascual-Leone A. Freitas C. Oberman L. Horvath J. C. Halko M. Eldaief M. (2011). Characterizing brain cortical plasticity and network dynamics across the age-span in health and disease with TMS-EEG and TMS-fMRI. *Brain Topogr.* 24 302–315. 10.1007/s10548-011-0196-8 21842407 PMC3374641

[B42] Rafiei M. H. Adeli H. (2017). A new neural dynamic classification algorithm. *IEEE Trans. Neur. Netw. Learn. Syst.* 28 3074–3083. 10.1109/tnnls.2017.2682102 28749358

[B43] Rossi S. Hallett M. Rossini P. M. Pascual-Leone A. (2009). Safety, ethical considerations, and application guidelines for the use of transcranial magnetic stimulation in clinical practice and research. *Clin. Neurophysiol.* 120 2008–2039. 10.1016/j.clinph.2009.08.016 19833552 PMC3260536

[B44] Rubinov M. Sporns O. (2010). Complex network measures of brain connectivity: uses and interpretations. *Neuroimage* 52 1059–1069. 10.1016/j.neuroimage.2009.10.003 19819337

[B45] Schmitt A. Hasan A. Gruber O. Falkai P. (2011). Schizophrenia as a disorder of disconnectivity. *Eur. Arch. Psychiatry Clin. Neurosci.* 261(Suppl. 2) S150–S154. 21866371 10.1007/s00406-011-0242-2 PMC3207137

[B46] Schwippel T. Schroeder P. A. Fallgatter A. J. Plewnia C. (2019). Clinical review: the therapeutic use of theta-burst stimulation in mental disorders and tinnitus. *Prog. Neuropsychopharmacol. Biol. Psychiatry* 92 285–300. 10.1016/j.pnpbp.2019.01.014 30707989

[B47] Shafi M. M. Pascual-Leone A. (2012). “Brain stimulation techniques and network studies of brain function,” in *Network Approaches to Diseases of the Brain*, eds Bianchi M. T. Cash S. S. Caviness V. S. (Sharjah: Bentham Science Publishers), 100–123. 10.2174/978160805017811201010100

[B48] Stanisz A. (2006). *Affordable Statistics Course (In Polish).* Cracow: Statsoft Polska.

[B49] Teneback C. C. Nahas Z. Speer A. M. Molloy M. Stallings L. E. Spicer K. M. (1999). Changes in prefrontal cortex and paralimbic activity in depression following two weeks of daily left prefrontal TMS. *J. Neuropsychiatry Clin. Neurosci.* 11 426–435. 10570754 10.1176/jnp.11.4.426

[B50] Valiulis V. Gerulskis G. Dapsys K. Vistartaite G. Siurkute A. Maciulis V. (2012). Electrophysiological differences between high and low frequency rTMS protocols in depression treatment. *Acta Neurobiol. Exp.* 72 283–295. 10.55782/ane-2012-1901 23093015

[B51] Van der Vinne N. Vollebregt M. A. van Putten M. J. A. M. Arns M. (2017). Frontal alpha asymmetry as a diagnostic marker in depression: fact or fiction? A meta-analysis. *Neuroimage Clin.* 16 79–87. 10.1016/j.nicl.2017.07.006 28761811 PMC5524421

[B52] Wilson D. L. (1972). Asymptotic properties of nearest neighbor rules using edited data. *IEEE Trans. Syst.* 3 408–421. 10.1109/tsmc.1972.4309137

[B53] Wilson M. T. Fulcher B. D. Fung P. K. Robinson P. A. Fornito A. Rogasch N. C. (2018). Biophysical modeling of neural plasticity induced by transcranial magnetic stimulation. *Clin. Neurophysiol.* 129 1230–1241. 10.1016/j.clinph.2018.03.018 29674089

[B54] Yuchao J. Mingjun D. Xi C. Jinnan G. Debo D. Jijun W. (2019). Aberrant prefrontal-thalamic-cerebellar circuit in schizophrenia and depression: evidence from a possible causal connectivity. *Int. J. Neural Syst.* 29:1850032. 10.1142/s0129065718500326 30149746

[B55] Zappasodi F. Marzetti L. Olejarczyk E. Tecchio F. Pizzella V. (2015). Age-related changes in electroencephalographic signal complexity. *PLoS One* 10:e0141995. 10.1371/journal.pone.0141995 26536036 PMC4633126

[B56] Zappasodi F. Olejarczyk E. Marzetti L. Assenza G. Pizzella V. Tecchio F. (2014). Fractal dimension of EEG activity senses neuronal impairment in acute stroke. *PLoS One* 9:e100199. 10.1371/journal.pone.0100199 24967904 PMC4072666

[B57] Zorzo C. Banqueri M. Higarza S. G. Pernia A. M. Arias J. L. (2019). Current state of transcranial magnetic stimulation and its use in psychiatry. *Actas. Esp. Psiquiatr.* 47 110–120. 31233209

[B58] Zrenner B. Zrenner C. Gordon P. C. Belardinelli P. McDermott E. J. Soekadar S. R. (2020). Brain oscillation-synchronized stimulation of the left dorsolateral prefrontal cortex in depression using real-time EEG-triggered TMS. *Brain Stimul*. 13 197–205. 10.1016/j.brs.2019.10.007 31631058

[B59] Zuchowicz U. Wozniak-Kwasniewska A. Szekely D. Olejarczyk E. David O. (2019). EEG phase synchronization in persons with depression subjected to transcranial magnetic stimulation. *Front. Neurosci.* 12:1037. 10.3389/fnins.2018.01037 30692906 PMC6340356

